# Genome-Wide Analysis of the *GDSL* Genes in Pecan (*Carya illinoensis* K. Koch): Phylogeny, Structure, Promoter *Cis*-Elements, Co-Expression Networks, and Response to Salt Stresses

**DOI:** 10.3390/genes13071103

**Published:** 2022-06-21

**Authors:** Yun Jiao, Jianhong Zhang, Cunde Pan

**Affiliations:** 1 Institute of Forestry, Ningbo Academy of Agricultural Science, Ningbo 315040, China; jydyx@163.com; 2 College of Forestry and Horticulture, Xinjiang Agricultural University, No.311 Nongda East Road, Urumqi 830052, China; pancunde@163.com

**Keywords:** genome-wide analysis, GDSL-type esterases/lipases, pecan, salt stress

## Abstract

The Gly-Asp-Ser-Leu (GDSL)-lipase family is a large subfamily of lipolytic enzymes that plays an important role in plant growth and defense against environmental stress. However, little is known about their function in pecans (*Carya illinoensis* K. Koch). In this study, 87 *CilGDSL*s were identified and divided into 2 groups and 12 subgroups using phylogenetic analysis; members of the same sub-branch had conserved gene structure and motif composition. The majority of the genes had four introns and were composed of an α-helix and a β-strand. Subcellular localization analysis revealed that these genes were localized in the extracellular matrix, chloroplasts, cytoplasm, nucleus, vacuole, and endoplasmic reticulum, and were validated by transient expression in tobacco mesophyll cells. Furthermore, the analysis of the promoter *cis*-elements for the *CilGDSL*s revealed the presence of plant anaerobic induction regulatory, abscisic acid response, light response elements, jasmonic acid (JA) response elements, etc. The qRT-PCR analysis results in “Pawnee” with salt treatment showed that the *CilGDSL42.93* (leaf) and *CilGDSL39.88* (root) were highly expressed in different tissues. After salt stress treatment, isobaric tags for relative and absolute quantitation (iTRAQ) analysis revealed the presence of a total of ten GDSL proteins. Moreover, the weighted gene co-expression network analysis (WGCNA) showed that one set of co-expressed genes (module), primarily *CilGDSL41.11*, *CilGDSL39.49*, *CilGDSL34.85,* and *CilGDSL41.01,* was significantly associated with salt stress in leaf. In short, some of them were shown to be involved in plant defense against salt stress in this study.

## 1. Introduction

The Gly-Asp-Ser-Leu (GDSL)-lipase family consists of hydrolytic enzymes widely distributed throughout prokaryotic and eukaryotic organisms. Some plant *GDSL* lipase genes have been successfully identified in various species, including 105 members in Arabidopsis (*Arabidopsis thaliana*) [1], 113 in rice (*Oryza* *sativa*) [2], 126 in white poplar (*Populus trichocarpa*), 57 in Physcomitrella (*Physcomitrella patens*), and others [3]. Gene structure (exon/intron) analysis in *Arabidopsis* shows that the *GDSL* lipase genes contain four introns (73 genes), three introns (11 genes), and two introns (12 genes) [4]. Alternatively, the intron numbers range from 0 to 26 in the *Rosaceae* GDSL esterase/lipase protein (GELP) genes [5]. Subcellular localization analysis using signal peptides identified 99 *Arabidopsis GDSL* lipase genes in the extracellular parts and organelles [6]. These different locations in the cell substructures mean that *GDSLs* may have a variety of biological functions. The structure of the rice GDSL esterase/lipase protein (OsGELP) is composed of six α-helices and six β-strands, but each member has distinct irregular loops and inserted sequences [7]. However, the crystal structure of plant lipases has not been reported to date.

*GDSL* lipases play the important roles in plant growth and development, resistance, morphogenesis, and pathogen defense [3,8,9]. For example, rapeseed (*Brassica napus*) *GDSL* lipase gene (*BnaLIP1*) was expressed in the roots, stems, leaves, flowers, fruit pods, flower buds, and other tissues [2]. Similarly, the co-expression and gene knockout analyses in *Arabidopsis* and *B. napus* demonstrate that *AtEXL6* and *BnEXL6* play a role in pollen growth and development [10]. Alternatively, plant hormone induction and regulation are required when certain *GDSL* lipases function in response to stress. Naranjo et al. found that the *Arabidopsis* lipase gene (*AtLTL1*) encoding the *GDSL*-motif under salt induction can enhance salt tolerance and also regulate pathogen resistance via salicylic acid activation [11]. Hence, salt stress can activate the *GDSL*-lipase gene, which plays a vital part in the plant’s defensive mechanism.

Pecan is one of the world’s most well-known dried fruit plants, and the fruits have high nutritional value [12,13]. The unsaturated fatty acid contents in the pecan kernel are higher than in olives, which is four-fold compared to walnuts (*Juglans regia*). Moreover, plants are known to be susceptible to a variety of abiotic factors that cause them to lose quality and yield and disrupt several biochemical and metabolic processes [14,15,16]. In the previous study, we enhanced the cultivation of pecans in coastal salt-affected soil near the beach, and the impact on pecan kernel quality was thoroughly studied. The findings revealed that when the concentration of NaCl in the fruit increased under salt stress, the fat content in the fruit gradually decreased, and the fatty acid composition and relative content also varied [17]. Furthermore, earlier research has demonstrated that GDSL lipases are involved in the synthesis and decomposition of fatty acids in plant seeds. It is a key enzyme in the metabolism of fatty acids that has been studied in model plants [18,19]. However, the pecan *GDSL*-type esterase/lipase genes have not been previously identified, and little is known about their regulatory mechanisms and responses to salt stress. In this study, the *GDSL*-type esterase/lipase genes were identified and analyzed in pecan, including their gene structures and protein physicochemical properties due to the importance of this gene family to dried fruit plants that primarily accumulate fatty acids. In addition, this study included a systematic evolutionary comparison analysis with model plants and an investigation of the changes in gene and protein expression levels with salt stress, providing a research basis for the regulation mechanism of GDSL esterase/lipase in pecan.

## 2. Materials and Methods

### 2.1. Plant Material and Sample Preparation under Salt Stress

The “Pawnee” pecan seedlings were collected at the three-leaf stage and transferred to a plastic hydroponic container (1000 × 40 × 28 cm, 15 plants per pot), with air pump ventilation containing a half-strength Hoagland solution. The solution was replaced weekly, and after 45 days of stable cultivation, the pecan seedlings were treated with variable concentrations of NaCl and collected for further experiments [20]. The seedlings were grown in hydroponic containers in the polyethylene (PE) greenhouse under natural light at 26 ± 3 °C air temperature and 75 ± 5% relative humidity (RH). The greenhouse uses a natural ventilation system to exchange the air inside and outside the greenhouse, and the PE greenhouse was isolated by gauze to avoid the spread of external pests. In detail, we collected the plants, which were divided into four groups, including the control group (no NaCl added), 0.3% NaCl treatment, 0.6% NaCl treatment, or 0.9% NaCl treatment for 24 h. Each group was done in triplicate, containing three leaves, three stems, and three roots. The collected samples were immediately frozen in liquid nitrogen for further analysis.

### 2.2. The GDSL-Type Esterase/Lipase Genes in the Pecan Genome and Subcellular Localization Analysis

The “Pawnee” pecan genome and protein sequences were downloaded from the *Juglandaceae* Plants database (http://www.juglandaceae.net/genome/cil/, accessed on 20 March 2021) [21]. The hidden Markov model (HMM) profiles of the GDSL-type esterase/lipase domain were then identified using the CLC Genomics Workbench software version 12.0 (QIAGEN, https://www.qiagen.com, accessed on 25 March 2021), based on the Pfam database (Pfam-A v32, http://pfam.xfam.org/, accessed on 17 May 2021). The physicochemical properties, including amino acid number, molecular weight, and isoelectric point (pI) of GDSLs, were predicted using ExPASy (https://web.expasy.org/protparam/, accessed on 16 July 2021). Additionally, subcellular localization was predicted with the WoLFPSORT online program (https://wolfpsort.hgc.jp/, accessed on 17 July 2021). Six *GDSLs* were randomly selected for transient expression analysis to validate the above-predicted results in silico. The specifics are as follows: a biotechnology company (Sangon Biotech, Shanghai, China) was directly commissioned to synthesize the *GDSL* coding sequence. Subsequently, infusion recombinase was used to insert the coding sequences into the pCAMBIA1300-GFP vector, resulting in the construction of a *GDSL*-GFP fusion gene driven by the CaMV-35S promoter, according to the relevant literature [22]. After transient expression in the leaves of *Nicotiana benthamiana* for two days, the GFP fluorescence signal was detected by using a Nikon C2-ER microscope (Nikon Corporation, Tokyo, Japan) equipped with a 488 nm excitation light, as previously described [23]. 

### 2.3. Phylogenetic Analysis, Gene Structure, and Cis-Acting Elements in GDSL Promoter Regions

The sequences of *GDSL*-type esterase/lipase genes from *Arabidopsis* were downloaded from the UniProt database (https://www.uniprot.org/, accessed on 22 July 2021). The identified *GDSL*-type esterase/lipase genes from pecan and *Arabidopsis* were then used to construct the phylogenetic tree using the CLC genomic workbench v 12.0 based on the neighbor-joining (NJ) method with 1000 bootstrap replicates. The phylogenetic tree was visualized in Figtree v1.4.3 [24]. 

The online MEME Suite (http://meme.nbcr.net/meme/intro.html, accessed on 28 July 2021) was used to analyze the protein sequences under the following parameters: The maximum number of motifs was 14. Furthermore, we first extracted the promoter sequences (the 2 kb sequences of the 5′ regulatory regions from the translational start sites) for every *GDSL* gene from the pecan genomic database and then submitted the sequences to the PlantCARE online tool (http://bioinformatics.psb.ugent.be/webtools/plantcare/html/, accessed on 30 July 2021) [25] to predict the *cis*-acting elements within the *GDSL* promoters. TBtools software (v1.0692) (https://github.com/CJ-Chen/TBtools, accessed on 29 July 2021) was used to visualize the conserved motifs and *cis*-acting element distribution. Phyre version 2.0 was used to look at the GDSL protein’s 3D structure as well. This tool can be found at: http://phyre2.phyre2.org (accessed on 30 July 2021).

### 2.4. Analysis of GDSL Expression Profiles Using qRT-PCR and Isobaric Tags for Relative and Absolute Quantification (iTRAQ)

The total RNA Trizol Extractor (Sangon, Shanghai, China) was used to extract the total RNA from each sample (root, stem, and leaf). Furthermore, the total RNA was digested with DNase I and reverse-transcribed using the M-MuLV First Strand cDNA Synthesis Kit (Sangon, Shanghai, China). Samples of cDNA were diluted to 1:5 with ddH_2_O for use in qRT-PCR. The gene-specific primers were designed using the OLIGO 7 analysis software [26] (Table S1). The qRT-PCR was run using an ABI Q6 (Applied Biosystems, Foster, CA, USA) with a 2× SG Fast qPCR Master Mix Kit (Sangon Biotech, Shanghai, China). The gene expression levels were calculated using the 2^−ΔΔCT^ method [27], including three independent technical and biological replicates to validate the qRT-PCR data. All qRT-PCR reactions were normalized with the housekeeping genes *CiActin* (GeneBank: NC-056755.1) and *β-Actin* (GeneBank: NM-007393) [28,29]. Finally, HemI 1.0.3.7 software (http://hemi.biocuckoo.org/down.php, accessed on 20 August 2021) was used to generate the gene expression heatmaps based on the qRT-PCR data. Additionally, as previously reported [30], the protein extracts of pecan leaves were used to isobaric tags for relative and absolute quantitation (iTRAQ) analysis. To undertake a functional annotation analysis of the DEPs selected for GDSLs, we used the NCBI website (https://www.ncbi.nlm.nih.gov/, accessed on 9 November 2021). The proteomics data from mass spectrometry have been submitted to the Proteome Xchange Consortium (http://proteomecentral.proteomexchange.org, accessed on 19 November 2021) under the dataset identifier PXD030031.

### 2.5. The Weighted Gene Co-Expression Network Analyses (WGCNA)

We performed WGCNA using the TBtools software (v1.0692) plugin-WGCNAshiny (https://github.com/CJ-Chen/TBtools, accessed on 26 May 2022), based on the expression levels from 87 *GDSL* genes in pecan with salt stress (as described above). The power of β = 12 (scale free R^2^ = 0.8) was selected as the soft-thresholding to ensure a scale-free network, with a minimum module size of 10. Module−trait associations were estimated using the correlation between the different tissues and the gene expression for salt treatments. The module with correlation coefficient >0.80 or <−0.80, and *p*-values <0.001, was defined as the sample-specific module. Finally, the co-expression networks were visualized through the OmicShare online tools (https://www.omicsshare.com/tools/, accessed on 26 May 2022) with a cut-off of the weight parameter obtained from the WGCNA set at 0.01. 

## 3. Results

### 3.1. Characterization of the GDSL-Type Lipase Genes in Pecan

**Figure 1:**
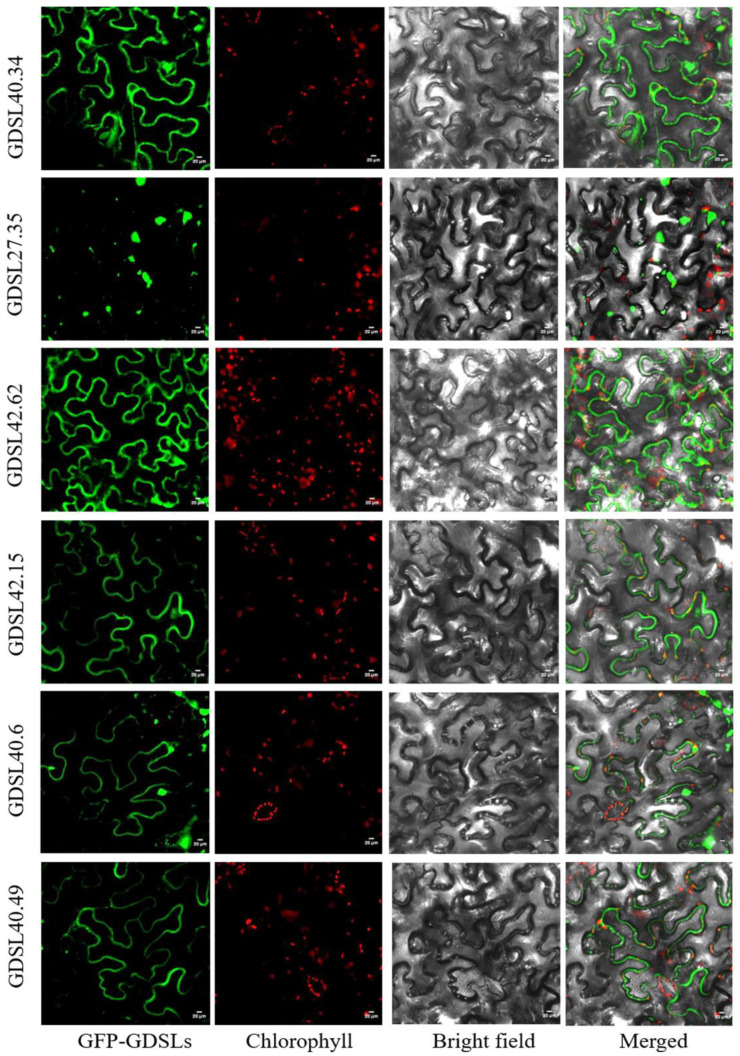
Subcellular localization of *GDSLs* in tobacco protoplasts. GFP signals were observed by confocal microscopy. Green represents green fluorescent protein (GFP) signals; red represents chlorophyll with autofluorescent light; gray represents bright field; merged images indicate the combined three channels. Similar results were obtained from three independent replicates, and representative images are shown. Bars = 20 mm.

Following GDSL domain verification using CLC workbench tools, 87 *GDSLs* were identified (Table S2). The proteins had a molecular weight (MW) ranging from 10.88 to 116.46 kDa, and these GDSLs were named after their molecular weights. The physicochemical properties of the *CilGDSLs* are presented in Table S2. The *GDSL* coding sequence (CDS) length ranged from 504 bp (CilGDSL18.29) to 3135 bp (CilGDSL11.65), with an average sequence length of 1084 bp. Among these, 48 (55.17%) *GDSL* lipase genes had four introns, 12 (13.79%) had five introns, six (6.90%) had three introns, and nine (10.34%) had only two introns. Of the CilGDSL proteins, CilGDSL11.65 was the largest with 1044 amino acids (aa), while CilGDSL10.88 was the smallest containing 98 aa. Moreover, the pI varied from 4.34 (CilGDSL39.16) to 9.44 (CilGDSL31.75). According to the instability index, 15 proteins may not be stable (>40, possibly not stable; ≤40, possibly stable). Subcellular localization was also predicted with the WoLFPSORT online program (Table S2). The results of subcellular localization showed that most of the genes were localized within the extracellular matrix (25 proteins) and chloroplasts (22 proteins). In addition, the proteins were mainly localized in the cytoplasm (15 proteins), nucleus (six proteins), vacuole (five proteins), endoplasmic reticulum (five proteins), etc. To verify the above predicted results in silico, six *GDSLs* were randomly selected for transient expression analysis. The results showed that *CilGDSL40.34*, *CilGDSL27.35*, *CilGDSL42.62*, *CilGDSL42.15*, *CilGDSL40.6*, and *CilGDSL40.49* were localized in the endoplasmic reticulum, cytosol, endoplasmic reticulum, plasma membrane, extracellular matrix, and plasma membrane of *Nicotiana benthamiana* leaves cells (Figure 1), respectively. Except for *CilGDSL40.49*, five *GDSLs* (83.33%) revealed subcellular localization trends that were similar across WoLFPSORT prediction and transient expression analyses (Table S2 and Figure 1). The WoLFPSORT prediction analysis was found to be very reliable as a result of these findings.

### 3.2. Phylogenetic Analysis and Gene Structure of 87 GDSLs

**Figure 2:**
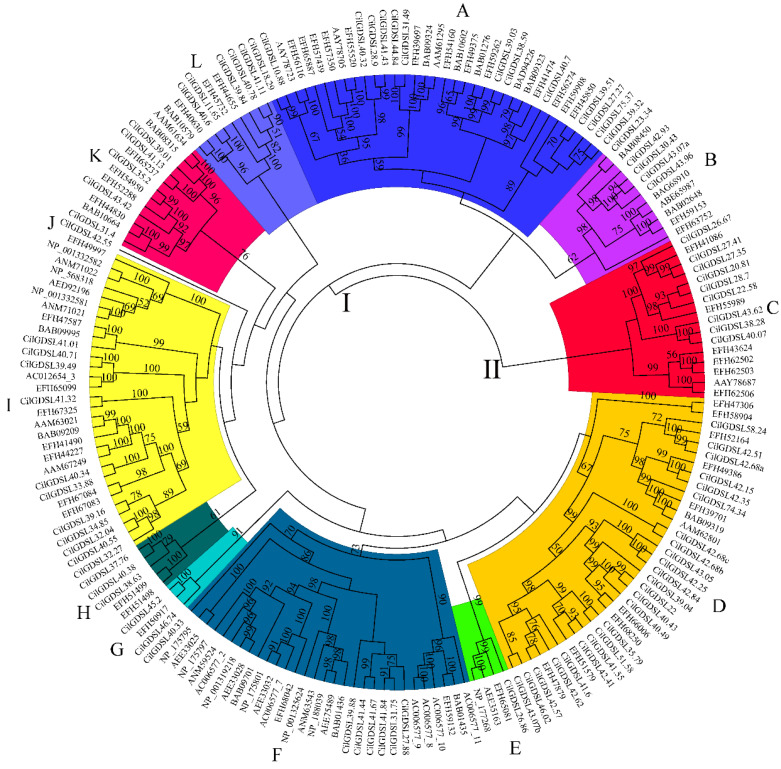
The phylogenetic tree of pecan and *Arabidopsis* GDSL proteins. The 12 subgroups (A–L) are denoted by a range of colors. The bootstrap value was calculated using 1000 replicates (only bootstrap values ≥50 were shown).

To explore the phylogenetic relationships of the GDSLs in pecan, the amino acid sequences of 191 GDSL proteins, including 87 from pecan and 104 from *Arabidopsis*, were aligned and used to construct an unrooted phylogenetic tree using the NJ method. These GDSLs were grouped into two groups (I and II) and 12 subgroups, including A (35 GDSLs), B (11 GDSLs), C (16 GDSLs), D (35 GDSLs), E (4 GDSLs), F (30 GDSLs), G (3 GDSLs), H (5 GDSLs), I (30 GDSLs), J (1 GDSL), K (12 GDSLs), and L (9 GDSLs) (Figure 2 and Table S3). It is worth noting that subgroup C was classified as a separate clade, group II, whereas the other subgroups were classified as a single large clade (group I), implying that subgroup C may have a unique protein structure. In general, the *GDSL* genes from *Arabidopsis* and pecan were scattered and clustered in different branches but did not form independent branches. The results showed that CilGDSLs may have different biological and molecular functions in different branches. 

**Figure 3:**
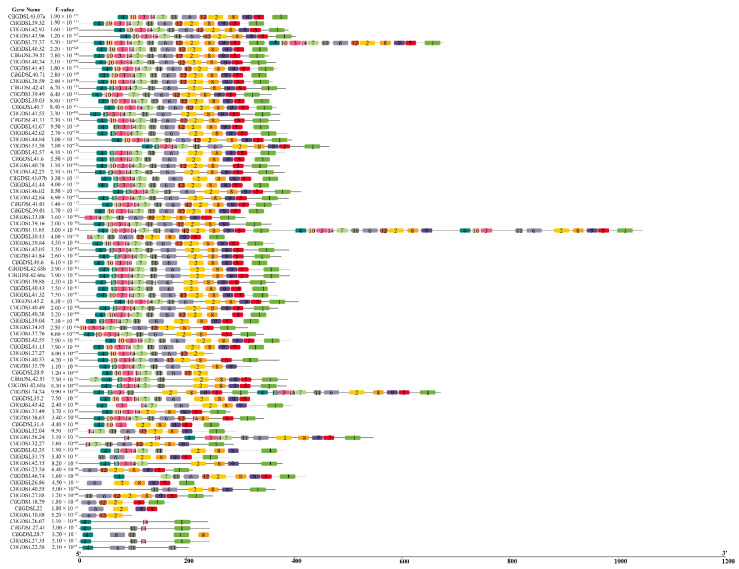
The distribution of conserved motifs among *CilGDSL* family members. The numbers in the colored boxes represent the 14 hypothetical patterns. All members’ names and cumulative E-values (<0.05) are displayed on the left side. The detailed information has been indicated in Table S4.

To ascertain the structural characteristics of GDSL proteins in pecans, two HMM profiles of GDSL proteins were identified using the CLC Genomics Workbench tools based on the Pfam database, including PF00657.22 and PF13472.6, which correspond to two conserved domains, Lipase_GDSL and Lipase_GDSL_2, respectively. Further, motifs of the 87 CilGDSLs were identified using the MEME online program (Figure 3), and 14 well-conserved motifs were detected (Table S4). The correspondence between the conserved domains and the conserved motifs was shown in Table S5. It can be seen that the Lipase_GDSL domain (PF00657.22) was the primary feature of GDSL proteins in pecans, which was found in 11 subgroups. In contrast, the Lipase_GDSL_2 domain (PF13472.6) was found exclusively in group C. Among them, *GDSL43.62* was the only gene that did not contain conserved motifs. Among the remaining 86 sequences, 59 contained more than 10 motifs, with most of them containing 12 or 13 motifs (49) and having similar structures, possibly leading to similar functions. In addition, there were three sequences with more than 20 motifs, namely *GDSL11.65* (34), *GDSL74.34* (21), and *GDSL75.35* (26) (Table S4 and Figure 3). The results of motif analysis were in line with the results of the phylogenetic analysis, which suggests that CilGDSLs with the same motifs in the subgroup may have the same functions.

**Figure 4:**
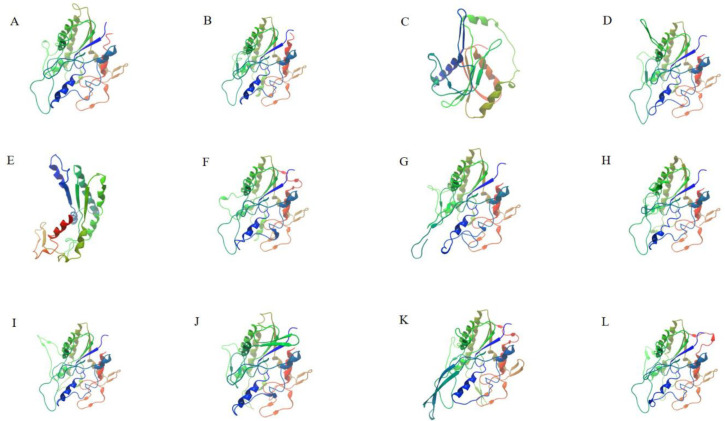
The tertiary structure of 12 representative predicted CilGDSL proteins in pecan. (**A**), CilGDSL44.84 (subgroup A); (**B**), CilGDSL43.96 (subgroup B); (**C**), CilGDSL43.62 (subgroup C); (**D**), CilGDSL42.68 (subgroup D); (**E**), CilGDSL26.86 (subgroup E); (**F**), CilGDSL39.88 (subgroup F); (**G**), CilGDSL46.74 (subgroup G); (**H**), CilGDSL38.63 (subgroup H); (**I**), CilGDSL39.49 (subgroup I); (**J**), CilGDSL42.55 (subgroup J); (**K**), CilGDSL43.42 (subgroup K); (**L**), CilGDSL39.84 (subgroup L).

The online protein homology recognition engine Phyre 2.0 was used to predict the tertiary structure of 87 CilGDSL proteins, with 97.4–100.0% confidence and 46–82% identity. The results showed that their tertiary structures were composed of different numbers in α-helices and β-strands. Based on the classification results of its genetic evolutionary relationship (Figure 2), a GDSL protein was selected from each subgroup that could represent the tertiary structural characteristics of the members of the subgroup, and its tertiary structure was recorded (Figure 4). The results showed that eight proteins (CilGDSL43.96, CilGDSL43.62, CilGDSL42.68c, CilGDSL39.88, CilGDSL46.74, CilGDSL38.63, CilGDSL39.49, and CilGDSL39.84) contained more than ten of the α-helices and β-strands. However, the remaining protein (CilGDSL43.42, subgroup K in Figure 3) contained four α-helices and the largest number (26) of β-strands, suggesting that its structure was more complicated and that it may perform different functions from the other proteins.

### 3.3. Detection of Cis-Acting Elements in Promoter Regions of CilGDSLs

**Figure 5:**
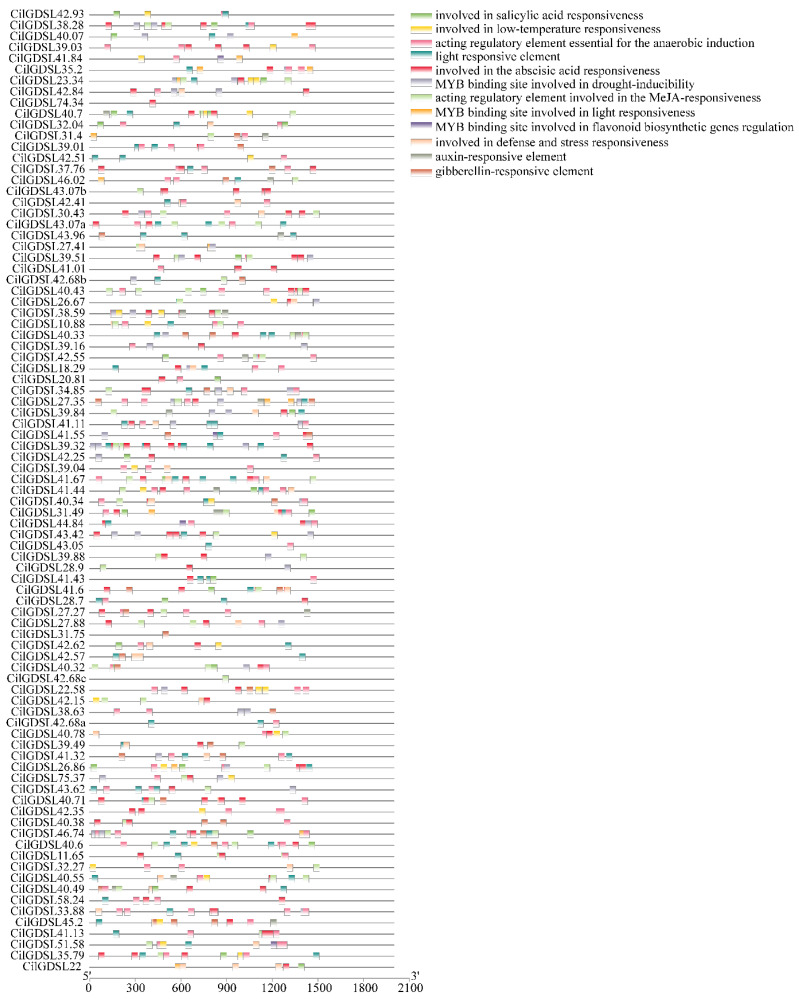
Predicted *cis*-elements in the promoter regions of *CilGDSL* genes. The names of the promoters are shown on the right side, and different colored squares represent different promoter elements.

According to the functional classification, a total of 12 main *cis*-acting elements were identified. The anaerobic induction regulatory elements and the action elements involved in the abscisic acid response were the most numerous in the *GDSL* gene promoter region, which were present in 68 and 66 *GDSL* gene promoter regions, followed by light response elements and jasmonic acid (JA) response elements present in 55 and 53 *GDSL* gene promoter regions (Figure 5). Taken together, MYB-related elements involved in drought response were detected in 40 *GDSL* genes. Three hormone-responsive regulatory elements were detected in the GDSL promoter region, and they were associated with gibberellin (GA), auxin (IAA), and salicylic acid (SA) responses. It is worth noting that only four *GDSL* genes (*CilGDSL41.84, CilGDSL41.55, CilGDSL44.84*, and *CilGDSL51.58*) have been identified with functional regulatory elements related to MYB flavonoid biosynthesis. In addition, components related to low temperature, defense, and pressure response were identified. Unfortunately, no elements associated with salt stress have ever been detected. In summary, the detection of the different types and numbers of regulatory elements in the *GDSL* gene promoter region indicates that *GDSLs* may be mainly involved in the response to environmental stresses such as light, drought, low temperature, and various hormone treatments.

### 3.4. Expression Profiles of GDSL Genes and iTRAQ Analysis in Response to Salt Stress

**Figure 6:**
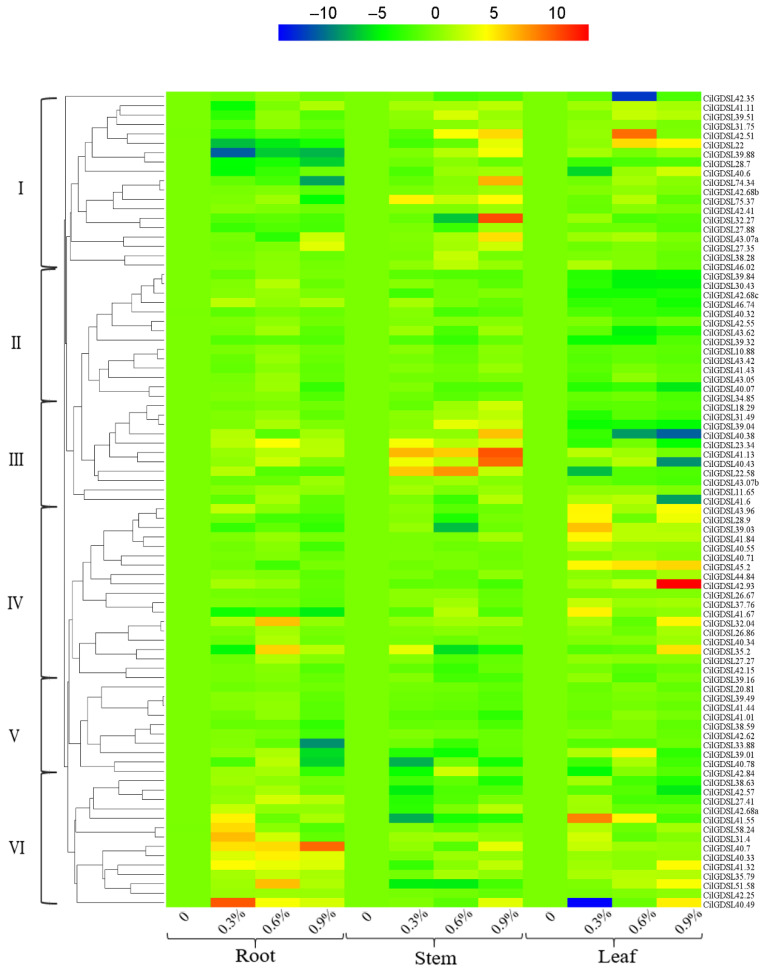
Heatmap of the *CilGDSL* gene expression patterns in pecan under salt stress conditions. *CilGDSL* gene expression levels were tested in the leaf, stems, and roots at the same time points (24 h) with different NaCl treatments (0, 0.3, 0.6, and 0.9%). The color scale depicts the degree of expression of each gene (log10) in a particular sample. The degree of up- or down-regulation is indicated by the intensity of red and blue colors, respectively.

The pecan “Pawnee” variety was used to conduct salt stress treatment experiments and investigate the tissue differential expression profiles of the 87 *GDSLs* by qRT-PCR and iTRAQ analysis (only leaves). According to the heatmap, the genes could be divided into six groups (Figure 6). Among them, the expression levels of *GDSLs* in group II had relatively small tissue differences. Only some of the *GDSLs* in groups V and VI had large changes in expression levels, such as *GDSL42.93* in the leaf and *GDSL39.88* in the root, showing a large increase and decrease, respectively. In addition, the expression levels of *GDSLs* in groups I, III, and VI had relatively large tissue differences, with a disordered expression pattern. After salt stress treatment, iTRAQ analysis revealed the presence of a total of ten GDSL proteins (Table 1). According to the phylogenetic evolutionary relationship tree (Figure 2), these proteins were mainly derived from subgroups L and F, as well as subgroups A, C, D, K, and I, with no GDSL proteins detected in other subgroups in response to salt stress. Among them, except GDSL40.88, all exhibited an up-regulated expression pattern; in addition, only four proteins (CilGDSL38.59, CilGDSL42.57, CilGDSL31.4, and CilGDSL27.35) were unable to be activated in the presence of 0.3% salt stress treatment (Table 1), indicating that they are not sensitive to lower concentrations of salt stress. Compared with the gene expression heat map of 87 *GDSLs*, there are certain differences, namely that some genes with significant expression differences did not appear in the iTRAQ analysis results. As a result, the *GDSLs* mentioned above responded to salt stress in different tissues and concentrations, suggesting that they may play different regulatory roles in different tissues and concentrations under salt stress.

### 3.5. Weighted Gene Co-Expression Network Analysis (WGCNA) of GDSLs in Pecan under Salt Stress

**Figure 7:**
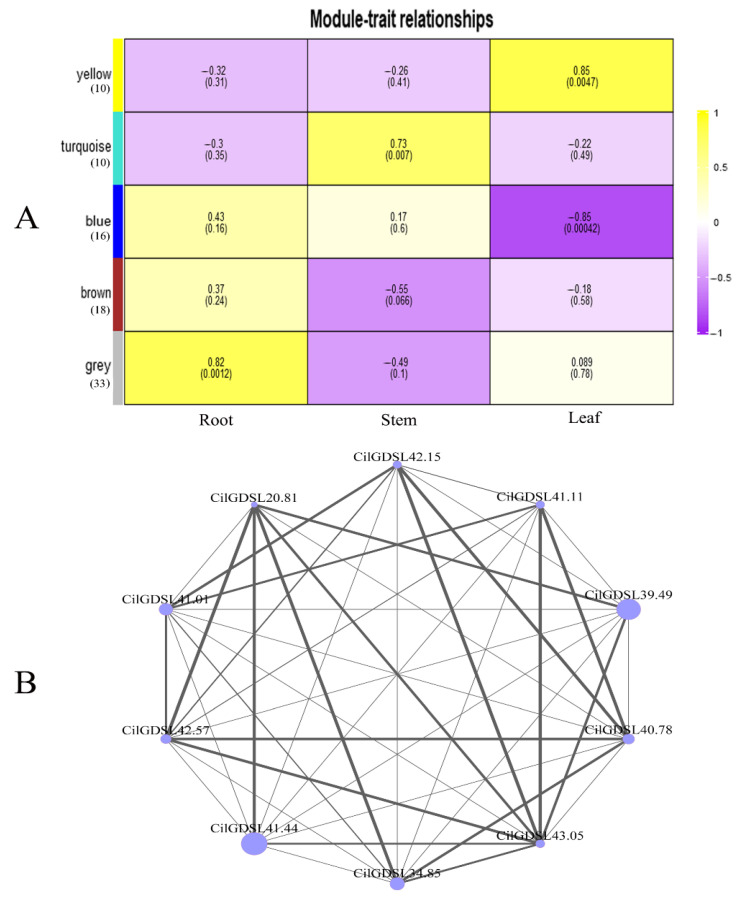
WGCNA analyses of *GDSL* genes and different tissues in pecan under salt stress conditions. (**A**), Heatmap of the correlation between the *GDSL* genes and different tissues in pecan under salt stress. Module trait relationship (*p*-value) for detected modules (*y*-axis, the number of genes in parentheses) in relation to different tissues (*x*-axis) for pecan. The module−trait relationships were colored based on the correlation between the module and the traits (the darker the color of the module, the more significant the relationship). The correlation coefficient was shown in each square and the *p*-value was listed in parentheses. (**B**), Interaction of the gene co-expression network of the *CilGDSL* hub genes in pecan under salt stress conditions. These genes are marked as node circles, and the size of the node circle is proportional to the edge weight. The line thickness indicates the strength of the relationship between two genes.

The weighted gene co-expression network was created to evaluate the association between the *CilGDSL* gene expression and different tissues for salt stress (Figure 7). First, WGCNA analysis of 87 *CilGDSL* genes produced five co-expression modules (Figure 7A), and one module was significantly correlated with phenotypes; the blue module was negatively associated with leaf tissue (module-trait correlations = −0.85, *p*-values = 0.00042) in response to salt stress. Next, the edges were screened by the criteria with a weight value of >0.01. The co-expression network was then visualized, and hub genes were identified using Omicshare’s online tools. Ten hub genes, including *CilGDSL41.11*, *CilGDSL39.49*, *CilGDSL34.85*, and *CilGDSL41.01*, were identified from the above module, as shown in Figure 7B. Additionally, the iTRAQ analysis revealed three DEPs (CilGDSL42.57, CilGDSL40.78, and CilGDSL41.11) with significant expression differences (Table 1), which also appeared in the co-expression network and as the hub genes. These key genes may play an important role in the leaf tissue for salt stress, which can be selected as the targets in future research.

## 4. Discussion

In this study, the results showed that only 87 *GDSL* genes were found in the pecan genome using the HMM profiles, which was less than the number of *GDSL*s in *Arabidopsis* (104) [1,7]. This could be related to the lower level of assembled genome in pecan (N50 scaffold length = 1.08 MB), which is a limitation. Previous studies have shown that most of the *GDSL* lipase genes in *Arabidopsis* are composed of four introns, consistent with the results of this study [2,31]. It is worth noting that the *GDSL* genes contained variable numbers of introns, including as high as 13 or as low as only one intron (Table S1). This variation may be caused by intron gain events. Related studies have shown that gains and losses of introns contribute to eukaryotic evolution [32,33]. These evolutionary events continue to occur, which may have a relatively large impact on the differences in gene structure and functional differentiation and may also be closely related to the occurrence and development of many biological processes and diseases. However, further exploration and verification are required in future studies of pecans. Many reports show that *GDSL* plays a vital role in plant immunity, particularly in rice and *Arabidopsis* [5,34,35]. In this study, phylogenetic analysis (Figure 2 and Table S3) demonstrated that *GDSL*-type esterase/lipase members *CilGDSL39.88, CilGDSL41.44, CilGDSL41.67, CilGDSL41.84,* and *CilGDSL31.75* are related to the *Arabidopsis* pathogen defense *GDSL* gene *GLIP4* (*BAB01436, AEE75489, NP_188039, ANM63543*, and *NP_001325624*). *GLIP4* (*BAB09701*) clustered with *CilGDSLs*, suggesting that they may play a role in pathogen defense also in pecans. Similarly, the pecan *GDSL* esterase/lipase member *CilGDSL26.86* and the *Arabidopsis* *GDSL* gene *GLIP6* (*EFH65081, AEE35163,* and *NP_177268*) formed an independent branch “E”. However, their specific functions have not yet been verified.

In addition, the subcellular localization of the *GDSL*-type esterase/lipase members in pecans was primarily restricted to the extracellular matrix and chloroplasts, suggesting that they mainly perform related functions at the above-mentioned positions, similar to the results of previous studies [7,36]. Related research results show that *GDSL* lipase in the extracellular matrix, together with proteins and carbohydrates, participates in the closure of leaf stomatal regulation pathways [37]. *Arabidopsis* *GDSL* lipase mutant *osp1* (*occluded of stomatal pore 1*) shows structural alterations to the stomatal cuticular ledge. In addition, they are necessary for leaf epidermal wax biosynthesis and stomatal cuticle formation to protect the underlying cells from radiation, drought, and pathogens [38,39,40]. This also provides an explanation and evidence for the localization of GDSL protein in the extracellular matrix. The bioinformatics analysis also demonstrated that the pecan *GDSL* members contained α-helix and β-strand structures as the primary components. This rule has also been found in the *GDSL* gene family of other plants. For example, the previous studies predicted the structures of multiple members of the rice *GDSL* gene family and found that the overall structure and folding pattern of the members are similar, but different members usually have specific irregular loops and inserted sequences [3]. These differences could change the protein folds and the active site, which could make different family members more or less capable of binding different types of substrates and having different biochemical functions. Previous studies have shown that *AtFXG1* modifies xyloglucan oligosaccharides through the hydrolysis of t-fucosyl residues [41]. Furthermore, our group’s previous analysis concluded that the up-regulation of L-fucose accumulation is a critical feature of pecans’ metabolic regulatory network under salt stress [30]. *CilGDSL42.57*, which is homologous to *AtFXG1*, exhibits the largest expression change in response to salt stress. Hence, it is suggested that *CilGDSL42.57* may be involved in the L-fucose metabolic process, which is linked to the pecan’s response to salt stress. Interestingly, *CilGDSL42.57* was also found in the co-expression network (Figure 7B) as a hub gene associated with salt stress in leaf tissue. There were nine co-expressed genes with it in the co-expression network, which could be its key upstream or downstream regulators involved in the regulation of salt stress and play an important role in the co-expression network. However, the functional annotation of these genes is still unclear, and genetic transformation is required to determine their precise roles. Furthermore, given the importance of many motifs in biochemical function, it is important to point out that motif-10, motif-12, and motif-13 are all very similar in pecan GDSL phylogeny and protein conformation grouping (Table S5), which allows the GDSL esterase/lipase to work with a wide range of substrates. As a result, these motifs should be further investigated to ascertain their roles in the molecular function diversification of *GDSLs* in pecans. 

Furthermore, 87 *cis*-acting elements in the promoter region of *CilGDSLs* were analyzed and identified. These results suggest that the expression of the above-mentioned *CilGDSLs* may be induced by hormones, chemicals, environmental stress, and pathogen infection. This is similar to earlier studies on *Sedum alfredii Hance* and grape berry (*Vitis vinifera* L.) [9,42]. It is worth noting that the novel findings in this study identified a large number of elements related to anaerobic induction. The waterlogging stress causes corn roots to be under a lot of pressure, which makes them less able to get the oxygen they need [43]. This causes changes in the expression of many genes that control lipid metabolism, glycolysis, energy metabolism, signal transduction, and photosynthesis. However, no detailed reports have linked the regulation and interaction between anaerobic induction and fatty acid metabolism. In addition, although related studies have demonstrated that some *GDSLs* are differentially expressed under salt stress [18,44], they have not yet found *cis*-acting elements related to salt stress in the promoter region of the *GDSL* genes, which is consistent with the results of this study. This may be due to these *GDSLs* indirectly responding to salt stress via distal promoters (beyond the detection region of the promoter in this study), or other promoters cooperating to induce pathways. However, these hypotheses need to be further verified.

According to the qRT-PCR analysis of 87 *CilGDSL* genes under salt stress conditions, different expression patterns in different salt stress concentrations and in different tissues and organs were detected (Figure 6). These results were similar to previously published reports [9,45]. For example, an early study by Cao et al. found that the *Arabidopsis* gibberellin delivery pathway regulates DELLA protein, down-regulating the expression of certain GDSL lipases in germinated seeds and closed flower buds [46]. The same gene showed opposite differential expression patterns in various tissues, even under the same salt stress concentration conditions, similar to *CilGDSL40.49* (0.3% NaCl treatment in roots and leaves). The positive and negative regulation modes indicated that the genes may play different roles in different tissues. In addition, *CilGDSL42.35, CilGDSL42.93, CilGDSL41.55, CilGDSL40.49, CilGDSL40.43*, and *CilGDSL41.6* were all up- or down-regulated in leaf tissue and belonged to the same branch “D”, according to the genetic evolution relationship (Figure 2); this suggests that they are specifically expressed in the leaf and have a role in salt stress and similar response mechanisms to related regulation. The qRT-PCR analysis results indicated that several *GDSLs* with high expression differences did not appear in the iTRAQ analysis, including CilGDSL27.88, CilGDSL40.38, CilGDSL42.93, and CilGDSL40.43 (Figure 6 and Table 1). Meanwhile, compared with the WGNCA analysis results, only three genes significantly associated with leaf tissue for salt stress appeared in the iTRAQ analysis results (Figure 7B and Table 1). Similarly, several studies reported a low correlation between mRNA and protein expression levels [47,48], suggesting that *GDSL* could have post-transcriptional modifications such as methylation or glycosylation in response to salt stress. Then, we only detected the expression of 10 GDSL proteins by iTRAQ analysis, implying that pecans respond to salt stress not mainly at protein levels or have the other more complex regulatory mechanisms, but still need further analysis and confirmation. In short, the pecan’s *GDSL*-type esterase/lipase genes and proteins respond to salt stress in a more complicated way, and they should be studied more to find out how they work and what their specific roles are through genetic transformation.

## 5. Conclusions

In this study, bioinformatic analysis of the *CilGDSL* gene family in pecan was carried out to identify the genetic structure, phylogenetic relationships, *cis*-elements in the promoter, and expression profiles of different tissues (roots, stems, and leaves), and salt stress. Some key expressed *CilGDSL* genes and proteins could be activated by salt treatment, according to qRT-PCR, iTRAQ, and WGCNA analysis, indicating that they may play a vital role in transcriptional and translational processes in the salt tolerance in pecans. This study will contribute to a deeper understanding of the pecan’s salt tolerance mechanism as well as a solid theoretical foundation.

## Figures and Tables

**Table 1:** The iTRAQ analysis of DEPs in pecan leaf following 24 h of salt stress compared to controls. * The gene codes in description that were derived from *Arabidopsis thaliana* (TAIR10) (https://www.arabidopsis.org/index.jsp).

No.	Gene	Protein Symbol	Description *	Log_2_ (Fold Change)			Subgroup
				0.3%	0.6%	0.9%	
1	CilGDSL41.11	LOC108993637	GDSL-like Lipase, At5g33370-like	1.28	1.37	1.27	L
2	CilGDSL39.84	LOC108993591	Li-tolerant lipase 1, LTL1-like	1.20	1.39	1.38	L
3	CilGDSL31.75	LOC108981353	GDSL-like Lipase, At5g03610-like	1.28	1.27	1.45	F
4	CilGDSL38.59	LOC108983791	GDSL esterase/lipase APG	-	1.38	1.37	A
5	CilGDSL42.57	LOC108982502	GDSL-like, AtFXG1-homologous	-	1.41	1.67	D
6	CilGDSL40.34	LOC108998072	GDSL-like Lipase, At5g45670-like	1.21	1.40	1.43	I
7	CilGDSL27.88	LOC108983860	GDSL-like Lipase, At5g03610-like	1.32	1.21	1.34	F
8	CilGDSL40.78	LOC108988817	GDSL-like Lipase, At5g33370-like	0.83	0.94	0.80	L
9	CilGDSL31.4	LOC109012662	GDSL-like Lipase, At4g28780-like	-	1.29	1.35	K
10	CilGDSL27.35	LOC108981001	SGNH hydrolase-type esterase, At5g62930-like	-	1.23	1.43	C

## Data Availability

Not applicable.

## Supplementary Materials

The following supporting information can be downloaded at: https://www.mdpi.com/article/10.3390/genes13071103/s1, Table S1: The primer sequences of 87 *GDSL* genes for qRT-PCR; Table S2: The list of identified *GDSL* members in this study; Table S3: The gene NAMEs of GDSL members from *Arabidopsis*; Table S4: Motif sequences identified by MEME tools; Table S5: Analysis of conserved motif in different groups of the phylogenetic tree.
