# Supplementary material for: Genome-Wide Analysis of the GDSL Genes in Pecan (Carya illinoensis K. Koch): Phylogeny, Structure, Promoter Cis-Elements, Co-Expression Networks, and Response to Salt Stresses
Source: Genes (Basel). 2022 Jun 21;13(7):1103. doi: 10.3390/genes13071103 (PMC9323844; doi:10.3390/genes13071103)
Supplement: Supplementary file 1 [file genes-13-01103-s001.zip › genes-1747095-supplementary.pdf]

Supplementary Materials:

Tables

Table S1 The primer sequences of 87 *GDSL* genes for qRT-PCR.

Gene Name	Forward Primer （5'-3'）	Reverse Primer （5'-3'）
CilGDSL42.35	TGTGCGGAGCTCCAAATGTA	AGATGTCACGGATGAGCCAAT
CilGDSL41.11	TGTCGGTGCCAATTTTGCT	ACTCGCCGCTGGTACTCTTG
CilGDSL39.84	ACTTTGCTTCTGCTGGGATTG	TAAGAGCGCTCACACGTTGCT
CilGDSL10.88	TTCCAGGTTACCACCAAATGC	GGGCAGGTATGCAGCCAAT
CilGDSL30.43	GATAAAGGGTGCTGCGGTACA	TGCTTCCGTGGGATGATAACT
CilGDSL43.96	CAGTGGAATTGCCCCCATAT	AAGTCGTTTGCAACAAAAACCA
CilGDSL43.07a	GATAAAGGGTGCTGCGGTACA	TGCTTCCGTGGGATGATAACT
CilGDSL38.63	GAGACTGGCACTGCTGATCCT	TCGGAGCTCGGCTTCCT
CilGDSL42.68a	GCTGGTCTGGGTTGGTGAAA	AATGACTGCAAAAAGTGGGTGAA
CilGDSL40.7	GGTCAGGTGAGGCAATTAGCA	TGCGAAGTTGCCCTTCATG
CilGDSL74.34	ACGGCATTACGGTTTTTTCAGT	GATGGATCTGCACAGGCTTTG
CilGDSL39.32	AAAGCCAAAGTTCCGGCAAT	ACCGGGAAAATCTCGACCATA
CilGDSL42.68b	TCGGGAAATCGCTCTACACAT	TGCCAGCAATTTGGGAAAC
CilGDSL40.07	TGGCCAAGACCGGTATCTTT	TCACACTGCTTGCAATTCGAA
CilGDSL38.28	ATTTTGGGTTGCTCCGTTGT	CGTATCGTGGACTTCCATCCA
CilGDSL26.67	ACACGTGCCTCTCGAAGAATATAA	CGCCCATCTTCATCAATTGG
CilGDSL40.71	TTCTTGGCCGCACAGTACAC	TGGGCTTTGAGCCAAGATG
CilGDSL40.32	TACCAGCTGGGATTGCGTACTA	ATTCTGTTCCTGCACGCATGT
CilGDSL18.29	GCTGGTCTTCCACCAATTGG	ATCGAAACCGTCCATGTTCTG
CilGDSL20.81	AATGGGTGTGAAGGTTGTCGAT	TCCCCAACAACCATCTTGCT
CilGDSL42.55	TGCTGCCTCCGGTATTCTTG	CGTCCCACTTGAGCTTCCA
CilGDSL42.68c	TCGGGAAATCGCTCTACACAT	TGCCAGCAATTTGGGAAAC
CilGDSL32.04	AGGATTCCATCCCACCCTATG	CACCCCGTGTTCTTCCAGAT
CilGDSL42.15	TTCGTGAGAATCCGGCTTTT	CGGCTGCCAGAGCTATGTAAT
CilGDSL41.13	AGGTGGTGGGATTCTGAATGAA	TCCTCTGGGCCAACTCTACTTT
CilGDSL43.42	GGAACAGCTCTCTTCTCAGTCACA	CATGGTGCCAACAAACACTTG
CilGDSL38.59	TTGGTGACTCTGCAGTGGATGT	CGTTGCAAAACCTCCCAGTAG
CilGDSL35.2	GGCCGATAGGCTGCATACC	GGGCGAGCAAATCCTTCA
CilGDSL39.88	TTTTGATCCAGGCAACAACCA	ACTAGACGGCCATCGGAGAGT
CilGDSL31.49	AGCAATTTTCCGCCATATGG	TGGCACATCCTCCTTTATACCAA
CilGDSL44.84	AGCATGGGACTTTGAGCAAGAA	TCCCATATGGCGGAAAATTG
CilGDSL39.16	GCAATGGCAGAACCATCGT	CGCCGGATGCATAATTCAC
CilGDSL39.49	AGACATCGCCGCTAATGCTAGA	CGGTTTCGACATGGTGTTTG
CilGDSL41.32	CTACTCGCGCATGCTAACTCAA	CGGCTGCCATTACCATGATA
CilGDSL40.78	CGAGACCAGCATGCTTTTTG	GATAGCGCTGAGGTTCATTGG
CilGDSL37.76	TGGGACTTCTGGGTGCATTC	AAGCATCGGGCTGCTATGAT
CilGDSL43.05	CTAGCCCTGCTGCCAACTTC	AGGTCTGTCCATTAGGCGGTTT
CilGDSL42.93	ATACAGCTGCCACCGAATGAA	CCCATAGGGAGGGAAATTGC
CilGDSL42.84	TGGGATTCAGGTGGCTCAGT	ATGTAAAGTGCCTGCCCAAAAT
CilGDSL22.58	AGCGAGGCCTCAGTTTGTTCT	CCTCGCAACACAATATCTGCTT
CilGDSL26.86	CTGCTGAAGGATGCACCTCTT	GACTGCATCGGGATACTTCCA
CilGDSL39.04	TCCTATCACTGGGTACGCTCATT	TTGATGCATACAGGGCTAGCA
CilGDSL51.58	ATTGGTTGCCTTCCCTATATTTG	AATGGCCTCCTTCAACTTGTGA
CilGDSL35.79	GATGGCCGGCTCACGAT	GGATGCAGACGTGGCAAAG
CilGDSL43.07b	GCGTACTGTTGTGGGCATGT	TGCGGCATCCCAACTAATG
CilGDSL46.02	ACGCCGTGGAACTCTTGGT	CGACGAAACGCTGCAGATT
CilGDSL42.41	TCCGGCCATCTTCAACTTTG	GAATACCGTCCTGCTGGAGAGT
CilGDSL42.51	CCATTTTCGTCTTCGCTTCTG	CGCTGATGGACTTGGTGTTG
CilGDSL75.37	GAAATGGCTGTCGCGAGAA	ATGGATTCGAAAACACCAACTTG
CilGDSL34.85	GGATGGGATGGAGCATGCT	CGATTCCGGCAAGGAGTTT
CilGDSL58.24	AATTGCTTCTCTAGGATTTTTGCTTT	CTGACTCGACGTGAAATGTGAAA
CilGDSL41.84	TCCGATGGCCGTTTGGTAT	CCCAGCAGATGCAAAATTCA
CilGDSL39.03	GCTAATCTCCAAAAGCAACTTCCT	GCAGCTTCTCCTTGCTTCCA
CilGDSL41.43	TCCTGCCATTCTCATCTTTGGT	GTCGGAATGTGATCAGGGAAA
CilGDSL28.9	TGTTCCGGACTTCATTGCAA	GATCCAGCTGAAGCAAAGCTAAC
CilGDSL45.2	GCCAACTTCATCAGCTCTTCAA	GTCGACGGAGGAGTCTCCAA
CilGDSL40.33	GAGTGCTCCCAAAAACCAATCA	TGCCTAGATAGCCACCCATGT
CilGDSL32.27	TTTGACCACTACATCCCACCATT	CCGATCTCCCGCAAATTGT
CilGDSL23.34	CCCAACCAAGCTGCAAAAAT	TAGATCAAGCAAAGGGCTGTATACA

CilGDSL28.7	GCTGTTCGGTTCTTCCATTGTT	GAAGTCCAGCCAGCGTATCC
CilGDSL41.55	TGGGTTTGAGGAGTCGTTGAG	GGCTTCCCCACCAAAACCT
CilGDSL41.44	TCCGATGGCCGTCTAATACC	TCCTGCTGATGCGAAGTTAGC
CilGDSL41.67	TGCTGCGGATCTGGTTCATA	TGTTGGATGAGCAGCGTCAA
CilGDSL46.74	CGGCTGAGAAGATGGGTTTG	TCTGGCTGCTCACGATTGTT
CilGDSL27.88	GCGTGCCGCCAATACTTTT	GGAGGGCCCTAGAAAGTGTGT
CilGDSL42.62	CCCACTTCCTGGCATTCTTG	GCATCAGGTAGCTGTTCCCTTAG
CilGDSL31.75	AGTTCCAAGTCTGCGAGAATCC	TGCTGTTGGTGATGCTTTCC
CilGDSL42.57	AGATGGCCGCCTCCTCAT	GATCCTCCGGTGGCAAAATT
CilGDSL22	CGTTTTAAGGCTCGCTCCATT	TGAATCAGCAAGGTCATTTTGC
CilGDSL40.55	CAAGTGTCGGAGCTGTTCACA	AGTAGCCGAAAATGCGATTTCA
CilGDSL41.01	GGCGCTGCACAGTGTAGGTT	TTGACATTGTCCACGCACCTT
CilGDSL40.43	TCTTCCGCACCCTGTTCTCT	TTTGGGAAGTTGACAGGGAAA
CilGDSL40.49	TCTTCCAAGGCCGGTTCTCT	TTGGGAAAATTGACAGGGAAAC
CilGDSL39.01	CCAATACATCGTGTTGCAAGGT	CTTTGTCCGTGGGATGGAAT
CilGDSL42.25	CCGATTCAAAGCTCGGGTTAT	AGATCATTCTGGCCTATGTCGAA
CilGDSL39.51	CTCGGAGCTCGGAAGATATGC	TTTGGCCACATTGTTGTATTCCT
CilGDSL43.62	TGGTGGGTGCCTTTCAAGTAC	GACGATTACCAAGTCAGCCAATG
CilGDSL27.27	CATCGGTGGATGCTGGAAAC	CAATTCGGCCATTGGAGAA
CilGDSL27.35	AATGCTCCCCCACGATGTT	TTGTCCTCTCTGGCAGTTCCA
CilGDSL11.65	TTGTTGACGGTGTGTGAAAAGTT	GCCGGAGTAACCAAAGATATCGT
CilGDSL40.6	GGCGACACAAGTAGGCAACTT	TCGTTGGTTCCAGTCACAATCA
CilGDSL41.6	ATAGCCGAGGAGCTGAAATCAC	ACTATACCCGCCTGGACGAA
CilGDSL40.34	GCGCTGGTTTTTGTGTTGAA	CAATGACTGGAGTTGGTTGTTATTG
CilGDSL33.88	GAGCTTTTGGGCTTCGATGA	TTGGCGCCCAGTTTCCT
CilGDSL27.41	GGCTTTGTTCTTGCTGCATCA	TGTTGTCGTTCGCTCGATCT
CilGDSL31.4	GTGGATGCTGGGAACAATGACT	GGTTCGACCATTGGTGAATCTT
CilGDSL40.38	ACAAGGAAAAAGAAGCACAACGT	GGAGCCATGGAGACTGGATGTA
CiActin	GCTGAACGGGAAATTGTC	AGAGATGGCTGGAAGAGG
β-Actin	ACCAACTGGGACGATATGGAGAAGA	TACGACCAGAGGCATACAGGGACAA

Table S2. The list of identified GDSL members in this study.

Name	Gene ID	ORF	Introns	AA	GDSL domain	MW	pI	Localization predicted	Instability index
CilGDSL42.35	CIL1317S0024	1146	4	381	42-365	42.35	5.71	extr	40.69
CilGDSL41.11	CIL1040S0027	1119	4	372	36-349	41.11	8.1	vacu, chlo, extr	33.07
CilGDSL39.84	CIL1040S0024	1089	4	362	27-339	39.84	5.46	E.R.	34.15
CilGDSL10.88	CIL0998S0036	282	1	98	3-94	10.88	5.12	cyto, chlo, nucl	33.90
CilGDSL30.43	CIL0937S0015	834	4	277	30-268	30.43	4.95	cyto, chlo	33.94
CilGDSL43.96	CIL0937S0017	1206	6	401	79-392	43.96	4.78	extr	31.28
CilGDSL43.07a	CIL0937S0016	1185	6	394	74-385	43.07	6.94	chlo	34.91
CilGDSL38.63	CIL1229S0052	1032	4	343	30-325	38.63	9.10	chlo	33.56
CilGDSL42.68a	CIL1229S0058	1158	1	385	43-364	42.68	8.25	cyto, nucl, plas	33.03
CilGDSL40.7	CIL0893S0343	1101	4	366	49-356	40.70	8.97	chlo, vacu	39.63
CilGDSL74.34	CIL0893S0324	2016	9	671	333-659	74.34	8.26	plas, golg, vacu	40.14
CilGDSL39.32	CIL1051S0084	1053	2	350	29-341	39.32	5.24	extr	36.22
CilGDSL42.68b	CIL0974S0084	1176	4	391	39-369	42.68	6.58	E.R., mito, plas	31.89
CilGDSL40.07	CIL0272S0013	1068	2	355	115-222	40.07	9.17	chlo, extr	30.08
CilGDSL38.28	CIL0272S0012	1035	2	344	104-206	38.28	8.68	nucl, cyto, chlo	38.64
CilGDSL26.67	CIL0987S0154	720	4	239	7-199	26.67	4.91	chlo, cyto	42.20
CilGDSL40.71	CIL1305S0039	1113	4	370	39-346	40.71	5.13	chlo, extr, vacu	28.97
CilGDSL40.32	CIL1152S0002	1089	2	362	35-348	40.32	6.38	vacu, extr	34.11
CilGDSL18.29	CIL1021S0080	504	2	167	3-156	18.29	5.06	cyto, chlo	38.61
CilGDSL20.81	CIL1021S0093	540	3	179	20-126	20.81	4.81	chlo	44.75
CilGDSL42.55	CIL1021S0036	1185	3	394	51-370	42.55	9.39	chlo, chlo_mito	26.19
CilGDSL42.68c	CIL1158S0011	1176	4	391	39-369	42.68	6.58	E.R., mito, plas	31.89
CilGDSL32.04	CIL0899S0135	876	4	291	8-268	32.04	8.88	nucl	29.82
CilGDSL42.15	CIL1207S0030	1134	4	377	41-365	42.15	7.90	Plas, extr	30.43
CilGDSL41.13	CIL1578S0001	1125	4	374	30-341	41.13	5.03	extr	35.48

CilGDSL43.42	CIL1092S0030	1188	4	395	36-376	43.42	8.55	vacu	27.65
CilGDSL38.59	CIL0993S0071	1080	4	359	37-350	38.59	8.67	chlo, extr	22.62
CilGDSL35.2	CIL0489S0002	972	4	323	42-308	35.20	8.42	extr	22.97
CilGDSL39.88	CIL1110S0011	1095	4	364	33-344	39.88	8.95	cyto, E.R.	27.03
CilGDSL31.49	CIL1086S0023	846	3	281	112-269	31.49	5.58	plas, golg	28.20
CilGDSL44.84	CIL1086S0024	1188	5	395	63-383	44.84	8.75	nucl, cyto	29.10
CilGDSL39.16	CIL1003S0121	1071	4	356	30-334	39.16	4.34	extr, vacu	29.05
CilGDSL39.49	CIL1265S0100	1074	4	357	26-333	39.49	8.41	-	27.95
CilGDSL41.32	CIL1265S0101	1110	4	369	35-348	41.32	8.99	extr	31.01
CilGDSL40.78	CIL1236S0054	1119	4	372	35-347	40.78	8.45	E.R.	37.82
CilGDSL37.76	CIL0909S0202	1032	4	343	11-326	37.76	5.26	extr, chlo	38.83
CilGDSL43.05	CIL1096S0013	1170	4	389	33-360	43.05	6.30	extr	31.08
CilGDSL42.93	CIL0001S0009	1167	4	388	68-379	42.93	5.92	golg, cyto	38.56
CilGDSL42.84	CIL0679S0003	1167	4	388	38-366	42.84	8.00	extr, chlo	26.93
CilGDSL22.58	CIL1204S0062	612	5	203	11-198	22.58	6.20	chlo, nucl, cyto	54.57
CilGDSL26.86	CIL1273S0056	711	1	236	2-212	26.86	6.04	cyto	23.35
CilGDSL39.04	CIL1075S0072	1056	4	351	15-332	39.04	7.98	cyto, chlo, mito	33.38
CilGDSL51.58	CIL1587S0002	1395	5	464	119-445	51.58	8.15	cyto, E.R.	44.57
CilGDSL35.79	CIL1587S0005	966	3	321	40-301	35.79	8.86	extr	36.53
CilGDSL43.07b	CIL0914S0113	1173	5	390	43-367	43.07	8.04	chlo, mito	37.62
CilGDSL46.02	CIL0914S0112	1239	4	412	65-390	46.02	8.67	E.R.	35.19
CilGDSL42.41	CIL0914S0120	1152	4	383	35-365	42.41	6.39	extr	35.50
CilGDSL42.51	CIL0908S0031	1164	1	387	44-366	42.51	7.57	chlo	33.86
CilGDSL75.37	CIL1293S0086	2034	5	677	30-342	75.37	8.20	extr, cyto	28.98
CilGDSL34.85	CIL1031S0087	942	4	313	22-288	34.85	9.27	nucl, cyto	20.58
CilGDSL58.24	CIL1526S0011	1641	1	546	198-520	58.24	6.92	chlo, nucl	78.41
CilGDSL41.84	CIL0427S0003	1128	5	375	39-352	41.84	9.42	chlo, extr	24.97
CilGDSL39.03	CIL0344S0022	1080	4	359	38-351	39.03	9.35	chlo, cyto, extr	30.17
CilGDSL41.43	CIL1114S0052	1107	2	368	45-359	41.43	7.95	extr, vacu	38.22
CilGDSL28.9	CIL1114S0050	783	1	260	45-260	28.90	6.73	extr	35.85
CilGDSL45.2	CIL1564S0012	1224	4	407	68-380	45.20	5.08	vacu	42.29
CilGDSL40.33	CIL1001S0040	1116	4	371	37-330	40.33	5.11	cysk, cyto	30.12
CilGDSL32.27	CIL1424S0030	864	4	287	9-267	32.27	6.65	cyto, cysk	43.28
CilGDSL23.34	CIL0649S0001	636	2	211	6-202	23.34	7.68	chlo	41.79
CilGDSL28.7	CIL1122S0052	768	5	255	11-197	28.70	5.41	chlo	49.52
CilGDSL41.55	CIL1043S0027	1122	5	373	23-353	41.55	8.82	extr	30.35
CilGDSL41.44	CIL1077S0026	1113	4	370	37-350	41.44	8.21	chlo	29.45
CilGDSL41.67	CIL1077S0025	1113	4	370	37-350	41.67	7.14	vacu	30.10
CilGDSL46.74	CIL1365S0006	1263	2	420	67-345	46.74	5.28	cyto, chlo	46.56
CilGDSL27.88	CIL1145S0003	747	3	248	10-229	27.88	9.41	chlo, chlo_mito	44.88
CilGDSL42.62	CIL1145S0155	1161	4	386	36-364	42.62	6.82	E.R.	37.93
CilGDSL31.75	CIL1145S0004	837	3	278	5-256	31.75	9.44	extr, chlo	32.79
CilGDSL42.57	CIL1145S0156	1149	4	382	35-363	42.57	6.75	extr	36.93
CilGDSL22	CIL1594S0025	585	5	194	10-161	22.00	9.21	chlo, nucl	34.81
CilGDSL40.55	CIL1436S0008	1095	4	364	136-343	40.55	5.98	chlo, golg	30.54
CilGDSL41.01	CIL0962S0082	1125	4	374	45-354	41.01	6.64	cyto, chlo	30.63
CilGDSL40.43	CIL0984S0106	1104	4	367	37-348	40.43	8.39	nucl	36.67
CilGDSL40.49	CIL1485S0011	1110	4	369	40-351	40.49	8.75	nucl	37.31
CilGDSL39.01	CIL0906S0036	1077	5	358	31-341	39.01	6.31	extr	32.30
CilGDSL42.25	CIL1055S0020	1146	4	381	32-359	42.25	5.99	extr, golg	39.01
CilGDSL39.51	CIL0942S0021	1059	2	352	30-342	39.51	4.68	extr, chlo	36.30
CilGDSL43.62	CIL1294S0004	1176	1	391	24-357	43.62	8.37	cyto, chlo, nucl	32.89

CilGDSL27.27	CIL1130S0009	747	1	248	37-244	27.27	5.75	extr	25.52
CilGDSL27.35	CIL1037S0017	729	5	242	5-204	27.35	5.55	cyto	50.07
CilGDSL11.65	CIL1417S0059	3135	13	1044	37-350	116.46	8.53	plas	34.21
CilGDSL40.6	CIL1417S0058	1101	4	366	30-347	40.60	8.90	extr, plas	21.89
CilGDSL41.6	CIL1118S0052	1125	4	374	35-352	41.60	8.27	-	36.00
CilGDSL40.34	CIL1084S0031	1098	4	365	33-345	40.34	5.60	E.R.	34.82
CilGDSL33.88	CIL1527S0002	927	4	308	12-288	33.88	4.91	chlo, nucl	30.98
CilGDSL27.41	CIL0938S0116	729	5	242	5-204	27.41	5.93	cyto, nucl	49.49
CilGDSL31.4	CIL0902S0109	843	4	280	98-258	31.40	6.51	cyto, extr	28.97
CilGDSL40.38	CIL1321S0025	1089	4	362	32-345	40.38	8.26	extr	37.65

Note: ORF, open reading frame; AA, amino acid; MW, molecular weight (kDa); pI, isoelectric point. Extr, extracellular matrix; vacu, vacuolar membrane; plas, plasma membrane; chlo, chloroplast; cyto, cytosol; nucl, nucleus; mito, mitochondrion; E.R., endoplasmic reticulum; golg, Golgi apparatus; cysk, cytoskeleton. Subcellular localisation was also predicted with the WoLFPSORT online webserver (https://wolfpsort.hgc.jp/). Gene ID were derived from pecan 'Pawnee' database ((http://www.juglandaceae.net/genome/cil/).

Table S3 The gene NAMEs of GDSL members from *Arabidopsis*.

Accession	Gene name
NP_001332582	GLIP7 GDSL-motif lipase 7, At5g15720, F14F8.100, F14F8_100
NP_001332581	GLIP7 GDSL-motif lipase 7, At5g15720, F14F8.100, F14F8_100
NP_001325624	GLIP4 At3g14225, MLE3.2
NP_175797	GLIP2 At1g53940, F15I1.2
NP_001319218	-
NP_175795	GLIP5 At1g53920, T18A20.15
NP_568318	GLIP7 At5g15720, F14F8.100
NP_188039	GLIP4 At3g14225, MLE3.2
NP_177268	GLIP6 At1g71120, F23N20.11
NP_175801	GLIP3 At1g53990, F15I1.7
ANM71022	GLIP7 GDSL-motif lipase 7, At5g15720, F14F8.100, F14F8_100
ANM71021	GLIP7 GDSL-motif lipase 7, At5g15720, F14F8.100, F14F8_100
ANM63543	GLIP4 GDSL-motif lipase 4, At3g14225
ANM59524	GLIP2 At1g53940, F15I1.2
AEE33028	-
AEE75489	GLIP4 At3g14225, MLE3.2
AEE35163	GLIP6 At1g71120, F23N20.11
AEE33032	GLIP3 At1g53990, F15I1.7
AEE33025	GLIP5 At1g53920, T18A20.15
AED92196	GLIP7 At5g15720, F14F8.100
AC012654_3	-
EFH68250	ARALYDRAFT_474829
EFH68042	GLIP3 ARALYDRAFT_337560
EFH67325	ARALYDRAFT_473537
EFH67084	ARALYDRAFT_473170
EFH67083	ARALYDRAFT_473169
EFH66006	ARALYDRAFT_334218
EFH65887	ARALYDRAFT_311764
EFH65237	ARALYDRAFT_895314
EFH65099	ARALYDRAFT_476288
EFH65081	GLIP6 ARALYDRAFT_476244
EFH63752	ARALYDRAFT_476497
EFH62506	ARALYDRAFT_319861
EFH62503	ARALYDRAFT_480827
EFH62502	ARALYDRAFT_319858
EFH59908	ARALYDRAFT_899264
EFH59262	ARALYDRAFT_479089, lipase APG
EFH59153	ARALYDRAFT_341606
EFH59132	ARALYDRAFT_897688
EFH58904	ARALYDRAFT_478320
EFH57439	ARALYDRAFT_482080
EFH57350	ARALYDRAFT_320775
EFH56274	ARALYDRAFT_903677
EFH56116	ARALYDRAFT_321717
EFH55989	ARALYDRAFT_903017
EFH55520	ARALYDRAFT_481942
EFH54950	ARALYDRAFT_481221
EFH54160	ARALYDRAFT_906695
EFH52288	ARALYDRAFT_348149
EFH52164	ARALYDRAFT_485206
EFH51579	ARALYDRAFT_322778, FXG1 alpha-fucosidase 1 homologous

EFH51499	ARALYDRAFT_484303
EFH51498	ARALYDRAFT_346891
EFH50917	ARALYDRAFT_489935
EFH49997	GLIP7 ARALYDRAFT_909546
EFH49386	ARALYDRAFT_487178
EFH49375	ARALYDRAFT_487160
EFH47879	ARALYDRAFT_909410
EFH47587	ARALYDRAFT_487685
EFH47306	ARALYDRAFT_487140
EFH45850	ARALYDRAFT_492123
EFH45732	ARALYDRAFT_491877
EFH44830	ARALYDRAFT_493795
EFH44655	ARALYDRAFT_915619, LTL homologous
EFH44227	ARALYDRAFT_492964
EFH43624	ARALYDRAFT_491739
EFH41490	ARALYDRAFT_49441
EFH41474	ARALYDRAFT_494383
EFH41086	ARALYDRAFT_496478
EFH40630	ARALYDRAFT_495588
EFH39701	ARALYDRAFT_916856
EFH39697	ARALYDRAFT_494382
AC006577_11	-
AC006577_10	-
AC006577_9	-
AC006577_8	-
AC006577_7	-
AC006577_2	-
ABE65987	At3g43550, T18D12.120
AAY78723	At2g40250, T07M07.13, T3G21
AAY78705	At2g30310, T9D9.12
AAY78687	At2g19010, F19F24.4, T20K24.2
AAM67249	At4g18970, F13C5.1
AAM63021	At5g45670, MRA19.6
AAM62801	At5g45910, K15I22.11
AAM61634	At5g37690, K12B20.17
AAM61295	At5g45960, K15I22.16
BAG68910	At1g58480 homologue
BAB02648	At3g14820, T21E2.10
BAB01436	GLIP4 At3g14225, MLE3.2
BAB01435	At3g14220, MLE3.1
BAB10664	At5g41890, K16L22.18
BAB10602	At5g22810, MRN17.4
BAB10579	At5g55050, K13P22.5
BAD94226	At5g45950, K15I22.15
BAB09995	At5g08460, F8L15.13, MAH20.2
BAB09701	GLIP1 At5g40990, MEE6.6
BAB09324	At5g45960, K15I22.16
BAB09323	At5g45950, K15I22.1
BAB09319	At5g45910, K15I22.11
BAB09209	At5g45670, MRA19.6
BAB08315	At5g37690, K12B20.17

Table S4 Motif sequences identified by MEME tools.

Motif	Length (aa)	Sequence
1	30	CSBPSKYVFWDGFHPTEAANKIIABQJLSG
2	29	ZFSNFIKELYGLGARKFVVHGLGPJGCLP
3	22	PTGRFSBGRLIIDFIAEALGLP
4	21	PAIFVFGDSLVDTGNNNYJNT
5	21	LISNPSKYGFEEPKKACCGTG
6	29	LGELPAKELLSKAJYLISIGSNDYLNNYY
7	21	GSNFLTGVNFASAGSGILNET
8	21	DGGGCVEEYNSAAQLFNRKLK
9	21	LRKELPGAKIVYVDVYSILYD
10	15	AKANYPPYGIDFPGG
11	15	VISLDKQJEYFKEYK
12	15	RSRQYTPPQYADFLI
13	10	PPYGETYFHR

Table S5 Analysis of conserved motif in different subgroups of the phylogenetic tree.

Subgroup*	Motif Number	Pfam accession	GDSL Domain
A	4-10-3-14-7-11-6-12-2-8-9-5-1	PF00657.22	Lipase_GDSL
B	4-10-3-14-7-11-6-12-2-8-9-5-1	PF00657.22	Lipase_GDSL
C	4-11-14-1	PF13472.6	Lipase_GDSL_2
D	4-13-3-14-7-11-6-2-8-9-5-1	PF00657.22	Lipase_GDSL
E	6-2-8-9-5-1-	PF00657.22	Lipase_GDSL
F	4-13-3-14-7-11-6-2-8-9-5-1	PF00657.22	Lipase_GDSL
G	4-7-11-6-12-2-8-9-5-1	PF00657.22	Lipase_GDSL
H	4-10-3-14-7-11-6-12-14-8-5-1	PF00657.22	Lipase_GDSL
I	4-10-3-7-11-6-12-2-8-9-5-1	PF00657.22	Lipase_GDSL
J	4-10-3-14-7-11-6-2-8-9-5-1	PF00657.22	Lipase_GDSL
K	4-3-14-7-6-2-8-9-1	PF00657.22	Lipase_GDSL
L	4-10-3-14-7-11-6-12-2-8-9-5-1	PF00657.22	Lipase_GDSL

Note: * The phylogenetic tree of pecan and *Arabidopsis* GDSL proteins (Figure 2). Motif sequences were shown in Table S4.
